# Inhibition of LPS-induced inflammation and signaling pathways in fallopian tube epithelial cells by estrogen and progesterone

**DOI:** 10.1186/s12917-025-04874-x

**Published:** 2025-07-02

**Authors:** Qianqian Li, Jianqi Liu, Bayaer Nashun, Sarina Bai, Xiaozhen Guo, Ling Wu, Tuya Bao

**Affiliations:** School of Basic Medicine, Inner Mongolia Medical University, Hohhot, 010110 China

**Keywords:** Estrogen, Progesterone, LPS, NF-κB, MAPK, PI3K/Akt

## Abstract

**Aim:**

The inhibitory effects of estrogen and progesterone on lipopolysaccharide (LPS)-induced inflammation and the nuclear factor-κB (NF-κB), mitogen-activated protein kinase (MAPK), and phosphoinositide-3 kinase (PI3K)/protein kinase B (Akt) signaling pathways in mouse oviductal epithelial cells were investigated.

**Methods:**

Mouse oviduct epithelial cells were isolated for various in vitro experiments. The CCK-8 method was used to detect the effects of LPS, LPS + Dexamethasone (DXM), LPS + E_2_, and LPS + P_4_ on the viability of fallopian tube epithelial cells. RT‒qPCR was used to measure the expression levels of IL-1β, TNF-α, IL-10, and β-defensin-2 (mBD-2) in cells. ELISA was used to measure the levels of IL-1β in cells. Western blotting was used to detect the protein expression of NF-κB p65, IκBα, p38, P-p38, Akt, and P-Akt in cells. Additionally, a salpingitis mouse model was constructed with LPS, and the model mice were treated with estrogen (E_2_), progesterone (P_4_), and DXM. The ovarian tissues were collected and subjected to HE staining. Moreover, IL-10 and mBD-2 expression in the tissue was detected by immunohistochemical staining.

**Key findings:**

Estrogen and progesterone significantly inhibited the production of IL-1β, TNF-α, IL-10, and mBD-2, thereby effectively suppressing the inflammatory response induced by LPS. In terms of signaling pathways, estrogen and progesterone significantly inhibited the protein expression of NF-κB, MAPK, and PI3K/Akt pathway members induced by LPS.

**Significance:**

Estrogen and progesterone can protect against LPS-induced mouse salpingitis by inhibiting the activation of the NF-κB, MAPK, and PI3K/Akt signaling pathways and suppressing the expression of the inflammatory factors IL-1β, TNF-α, IL-10, and mBD-2.

**Brief Summary:**

Estrogen and progesterone can effectively reduce the inflammatory response of the fallopian tubes.

**Strengths and limitations of this study:**

The advantages of this study are as follows: high data reliability; a large number of experimental repetitions, resulting in stable outcomes; and a concise and efficient explanation of the issues.

**Limitation:**

The research objective is too broad.

**Supplementary Information:**

The online version contains supplementary material available at 10.1186/s12917-025-04874-x.

## Introduction

Infertility is a medical condition defined as the inability to become pregnant after a year or more of regular, unprotected sexual intercourse or after artificial insemination. Approximately 15% of couples in the United States are affected by infertility [1]. Female infertility accounts for approximately 50% of all infertility cases, and tubal diseases are responsible for 10-30% of female infertility [2]. The WHO predicts that infertility may become the third-most common disease affecting human health after cancer and cardiovascular diseases in the 21st century.

Salpingitis is a serious reproductive tract disease, and its sequelae include tubal blockage, infertility, ectopic pregnancy, and chronic pelvic pain. The fallopian tubes of mammals play an important reproductive role in the transportation of eggs, transportation of sperm, activation of sperm, and early embryonic development [3]. The normal epithelial cells of the fallopian tubes secrete fallopian tube fluid, which can provide nutritional support for sperm and early embryos, and also helps with the self-defense of the fallopian tubes [4]. Recent studies have shown that tubal inflammation can cause various cytotoxic factors and enzymes to be released into the fluid inside the fallopian tubes, leading to fertilization disorders, early embryo growth defects, and abnormal transport and implantation of fertilized eggs, ultimately resulting in pregnancy failure [5]. Salpingitis is a clinically common and difficult-to-treat disease with various causes and presents multiple inflammatory patterns. It can be classified into acute salpingitis and chronic salpingitis on the basis of the course of the disease. The etiology of this disease can be divided into nonspecific salpingitis and specific salpingitis. The main clinical triggers include pathogen infections, iatrogenic infections, the spread of inflammation from adjacent organs, and high-risk sexual behaviors, among others. The main research topics include eosinophilic salpingitis related to parasitic infections [6], nodular salpingitis associated with infections, cellular invasion, congenital malformations [7], and salpingitis related to *Chlamydia trachomatis* infection [8]. Salpingitis may lead to obstruction of the fallopian tubes, which can result in infertility.

Many previous studies have shown that estrogen (E_2_) and progesterone (P_4_) have anti-inflammatory effects. Estradiol is the most potent natural estrogen in the human body and plays a key role in reproductive health and metabolic regulation. According to the relevant literature, estradiol can effectively inhibit lipopolysaccharide (LPS)-induced inflammatory responses in ovine fallopian tube epithelial cells by downregulating the p38 and NF-κB signaling pathways [9]. Estrogen can significantly reduce the upregulation of inflammatory factors such as IL-1β, IL-6, and TNF-α in LPS-induced rat uterine epithelial cells [10]. Additionally, a decrease in estradiol can lead to an increase in the expression of inflammatory factors such as TNF-α and IL-6 [11]. In addition to estradiol, progesterone is also an important steroid hormone that is primarily secreted by the corpus luteum in female ovaries and plays a central role in reproductive health, maintenance of pregnancy, and endocrine regulation. Previous experiments have shown that progesterone can effectively inhibit the production of inflammatory factors in LPS-induced bovine endometrial epithelial cells by intervening in the NF-κB and MAPK signaling pathways [12]. Furthermore, progesterone effectively suppressed the expression of inflammatory factors in LPS-induced human fetal membrane cells [13]. Therefore, on the basis of previous experiments, the anti-inflammatory effects of E2 and P4 as well as their possible molecular mechanisms were investigated in this study through in vitro and in vivo experiments.

## Materials

### Animals and cell lines

Thirty female Kunming mice (6–8 weeks old; 18–22 g) were provided by the Experimental Animal Center of Inner Mongolia Medical University (Inner Mongolia Autonomous Region, Hohhot City). The animal experiments were approved by the Animal Ethics Committee. These animals were kept in plastic cages with a controlled temperature of 20–26 °C and humidity of 50–60% under a 12 h/12 h light/dark cycle. The fallopian tube epithelial cells were isolated from the mice (Procell, Wuhan).

### Main reagents and instruments

Mouse fallopian tube epithelial cells were cultured with complete culture medium, fetal bovine serum, antibiotics, DMSO, polylysine, trypsin, and PBS (Procell, Wuhan). Collagen type I (Sigma), polyformaldehyde (China National Pharmaceutical Group Corporation), concentrated normal goat serum (Wuhan Boster Biological Technology), Cy3-labeled sheep anti-mouse IgG (Wuhan Boster Biological Technology), Triton X-100 (Beyotime), DAPI (Beyotime), fluorescence quenching sealing agent (SouthernBiotech), and a CCK-8 assay (Biosharp) were purchased.

## Methods

### CCK-8 assay to evaluate cell proliferation

The effects of LPS, LPS + Dexamethasone (DXM), LPS + E_2_, and LPS + P_4_ on the viability of fallopian tube epithelial cells were evaluated via the CCK-8 method. The epithelial cells of the fallopian tube were seeded at a density of 1 × 10^5^ cells per well in a 96-well plate for 24 h, treated with different concentrations of DXM, E_2_, and P_4_ in 100 µL, and incubated for an additional 12 h in the incubator. Then, 10% CCK-8 was added to each well, and the samples were incubated for 2 h. The optical density (OD) was measured at 450 nm via a microplate reader. The percentage of viable cells was calculated using the following formula: (OD treatment - OD blank)/(OD control - OD blank) × 100%.

### RT‒qPCR detection of the expression levels of IL-1β, TNF-α, IL-10 and β-defensin-2 (mBD-2) in cells

The cells were collected, total RNA was extracted according to an RNA extraction manual, reverse transcription was performed, and the samples were incubated at 42 °C for 2 min to remove genomic DNA. The mixture was incubated at 37 °C for 15 min and then at 85 °C for 5 s. The specific primer sequences are shown in the table below, with β-actin as the internal reference. The primers were synthesized by Sangon Biotech. The reaction conditions for real-time PCR were as follows: predenaturation at 95 °C for 30 s, denaturation at 95 °C for 5 s, and annealing at 60 °C for 30 s for a total of 40 cycles. The Ct values of each experimental group were measured separately, and the relative expression levels of the target genes were calculated via the ΔΔCt method. The primer sequences are shown in Table 1.

**Table 1:** Primer sequences

Target gene	Forward primer (5’-3’)	Reverse primer (5’-3’)
β-actin	GATTACTGCTCTGGCTCCTAGC	GACTCATCGTACTCCTGCTTGC
IL-1β	CAACCAACAAGTGATATTCTCC	GATCCACACTCTCCAGCTGCA
IL-10	TGCTAACCGACTCCTTAATGCAGGAC	CCTTGATTTCTGGGCCATGCTTCTC
TNF-α	GCACCACCATGAAGGACTCA	TCGGAGGCTCCAGTGAATTCG
mBD-2	TCTCTGCTCTCTGCTGCTGATATGC	AGGACAAATGGCTCTGACACAGTACC

### ELISA detection of IL-1β levels

After treating the cultured mouse fallopian tube epithelial cells according to the intervention protocols, and the supernatant was collected for testing. First, blank wells and samples wells were set up on the ELISA plate. Next, 40 µL of sample mixture was added to the wells containing the test samples, and then 10 µL of the test sample was added. One hundred microliters of enzyme-labeled reagent was added to each well, excluding the blank well, and the plate was sealed with sealing film and incubated at 37 °C for 60 min. The mixture was discarded, the wells were shaken dry, each well was filled with wash solution, and the wash solution was discarded after 30 s. Then, 50 µL of color developer A was added to each well, followed by 50 µL of color developer B. The mixture was gently shaken, and incubated at 37 °C in the dark for 15 min. Fifty microliters of stop solution was added to terminate the reaction. The OD values of each well were measured at a 450 nm wavelength, and the IL-1β levels were calculated.

### Western blot detection of NF-κB p65, IκBα, p38, P-p38, akt, and P-Akt protein expression

RIPA lysis buffer was used to extract total protein from fallopian tube epithelial cells. The protein concentration was quantified using a BCA assay kit. Equal amounts of protein were separated via 10% SDS‒PAGE and then transferred to a PVDF membrane. In accordance with our previous protocol, the PVDF membrane was blocked with BSA and then treated with primary and secondary antibodies. The signal intensity was quantified via a chemiluminescence detection system.

### Establishing a mouse model of ovarian ablation

A total of 0.15 ml of 1% pentobarbital sodium was injected into the abdominal cavity of each mouse. After 3 min, when the mouse was in an anesthetized state, the mouse was placed on its back, the back fur was shaved, the area was disinfected, and a horizontal incision of 1–2 cm near the rib arch on the back of the mouse was made. The fat on both sides of the ribs was removed with forceps, the germinal epithelium was separated, and the ovaries inside were removed. The uterine horn was placed back into the abdominal cavity with forceps, and the abdominal cavity was closed. In the sham surgery group, only the fat tissue around the ovaries was removed, and the remaining procedures were the same.

### Grouping and intervention

Two weeks postsurgery, the mice were randomly divided into four groups: the sham surgery group (SHAM), the ovariectomy group (OVX), the ovariectomy plus estrogen group (OVX + E_2_), and the ovariectomy plus progesterone group (OVX + P_4_), with 5 mice in each group. Each group of mice received intraperitoneal injections once a day according to the required drug dosage, with each group receiving 0.1 mL of the drug for one week. See Table 2.

**Table 2:** Intervention protocols for the mice (*n* = 5)

Group	Handing method	Medicine	Dosage	Time(d)	Route of administration
SHAM	Fake surgery	Vegetable oil	0.1mL/d	7	Abdominal cavity injection
OVX	Bilateral oophorectomy	Vegetable oil	0.1mL/d	7	Abdominal cavity injection
OVX + E2	Bilateral oophorectomy	Estrogen	50 µg/kg/d	7	Abdominal cavity injection
OVX + P4	Bilateral oophorectomy	Progesterone	100 mg/kg/d	7	Abdominal cavity injection

### Hematoxylin and Eosin (HE) staining of tissues

The pathological damage to tubal tissue induced by LPS was evaluated through histopathological examination. The tubal tissues from each group of mice were collected, fixed in 10% formaldehyde for 24 h, dehydrated in ethanol, embedded in paraffin after clearing with xylene, cut into 0.5 μm thick sections with a microtome, and then stained with HE. In accordance with the requirements of the experimental design, different researchers have observed histopathological lesions via optical microscopy.

### Immunohistochemical staining was used to detect the expression levels of IL-10 and mBD-2 in tissues

The fallopian tubes were collected from mice in each group, fixed, dehydrated, embedded in paraffin, and processed into 3 μm thick sections. The paraffin-embedded sections were deparaffinized with xylene, immersed in 3% H_2_O_2_ at room temperature for 10 min, and washed with PBS for 5 min. The tissue sections were placed in antigen retrieval solution and microwaved at a high temperature for 15 min. The endogenous peroxidases in the area of interest were blocked with a blocking solution at room temperature for 10 min, and the samples were washed with PBS three times. The samples were incubated with primary antibodies according to the instructions and washed with PBS three times. Then, a biotinylated goat anti-mouse IgG was added, the samples were incubated at room temperature for 10 min, and the samples were washed with PBS three times. A horseradish peroxidase solution was incubated at room temperature for 10 min. After color development with DAB, the sections were washed with PBS, counterstained with hematoxylin for 1 min, cleared with xylene, dehydrated, mounted on slides and coverslipped with neutral gum, and observed and photographed under a light microscope. Positive expression of the IL-10 and mBD-2 proteins was indicated by the appearance of corresponding brown deposits in epithelial cells. Analysis method 1: The optical density integral was measured via ImageJ software. Analysis method 2: The tissues were scored based on the proportion of positive cells and the staining intensity in the high-power field (200×). The staining intensity and positive cell proportion scores were added to obtain the immunohistochemistry score. The scoring process strictly adheres to experimental blind methods, with different personnel conducting the scoring for different groups. In the case of differing opinions, a third party evaluated the scores.

### Statistical methods

Data analysis was performed using GraphPad Prism 8.0. All the data were calculated as the mean ± standard error of the mean (SEM) from three independent experiments. Statistical analysis was conducted using one-way analysis of variance and Dunnett’s multiple comparison test. A p value < 0.05 was considered to indicate statistical significance.

### Results

### Morphological observation of fallopian tube epithelial cells

**Fig. 1:**
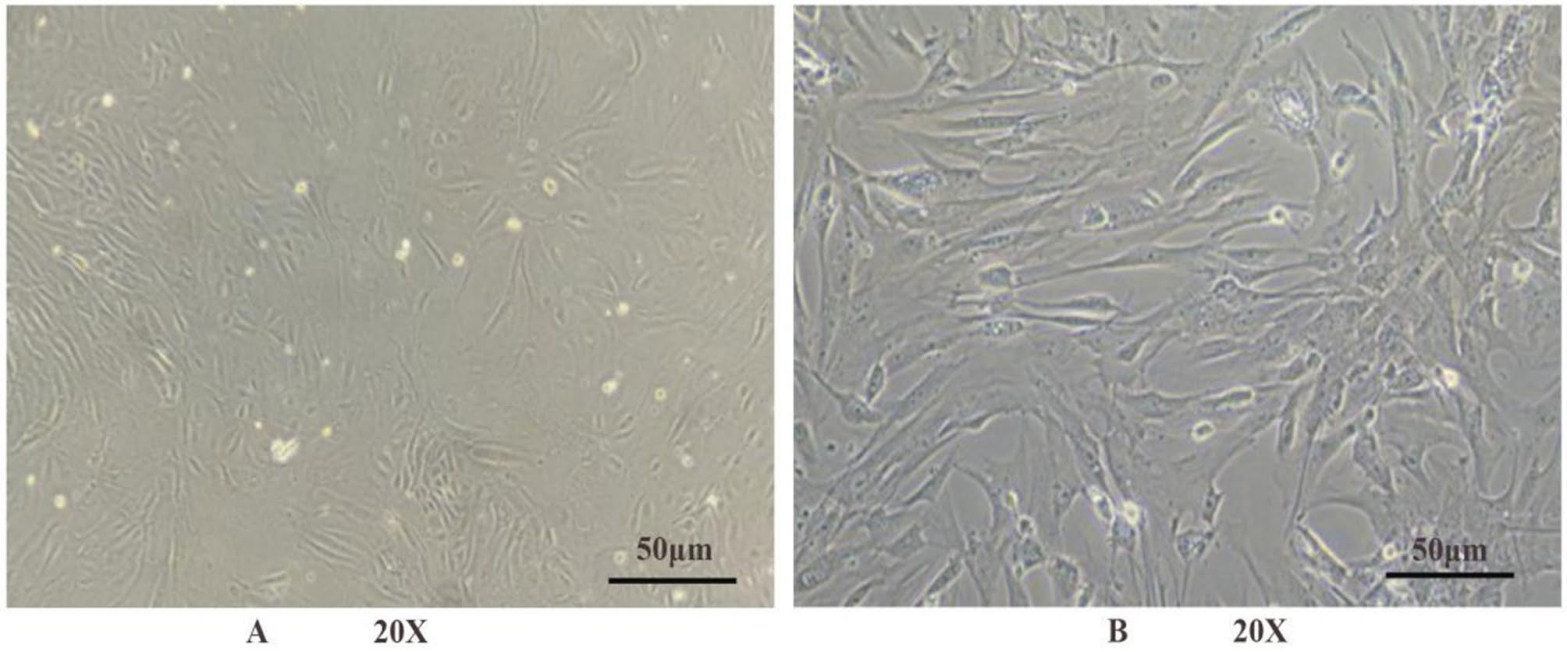
Morphology of mouse fallopian tube epithelial cells

The mouse fallopian tube epithelial cells used in this study were purchased from Wuhan Puno Sai Life Science Technology Co., Ltd. Before proceeding with subsequent experiments, the cell status was verified via an optical microscope. After 24 h of culture, the cells exhibited diverse morphologies, including flattened oval, polygonal, and elongated spindle shapes. After 5 days of culture, the cells had reached 80-90% confluence, and their morphology became uniform, appearing elongated and slightly smaller in size (Fig. 1).

### CCK-8 assay to evaluate cell proliferation

#### Effects of different concentrations of LPS on the proliferation of mouse oviduct epithelial cells

**Fig. 2:**
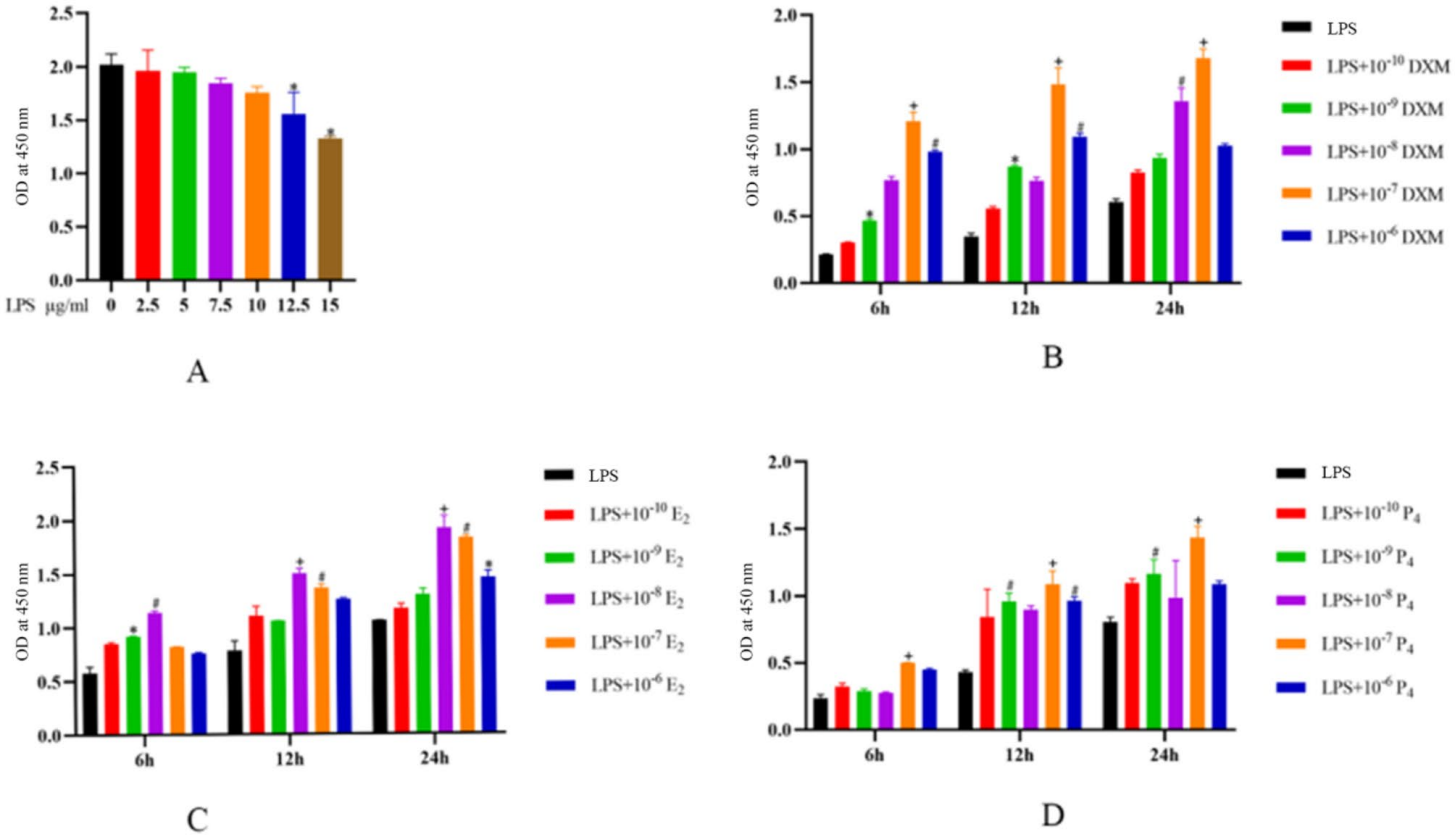
CCK-8 assay to evaluate cell proliferation **A**: Effects of different concentrations of LPS on the proliferation of mouse oviduct epithelial cells. **B**: Effects of different concentrations of DXM on the proliferation of mouse oviduct epithelial cells. **C**: Effects of different concentrations of E_2_ + LPS on the proliferation of mouse oviduct epithelial cells. **D**: Effects of different concentrations of P_4_ + LPS on the proliferation of mouse oviduct epithelial cells. **Note**: Compared with the control group, *P < 0.05, #*P* < 0.01, +*P* < 0.001

Compared with those of the control group, the OD values of the 0, 2.5, 5, 7.5, and 10 µg/mL LPS groups did not significantly differ (*P* > 0.05), whereas the OD values of the 12.5 and 15 µg/mL LPS groups were significantly decreased compared with those of the control group (*P* < 0.05) (Fig. 2 - A).

#### The combined effects of DXM and LPS on the proliferation of mouse oviduct epithelial cells

The CCK-8 assay results revealed that, compared with those of the LPS group, the OD values of the DXM groups were greater. Compared with that of the LPS group, the OD value of the LPS + 10^− 7^ mol/L + DXM group was significantly greater (*P* < 0.001) (Fig. 2 - B).

#### The combined effects of E_2_ and LPS on the proliferation of mouse oviduct epithelial cells

The CCK-8 assay results revealed that, compared with those of the LPS group, the OD values of the E_2_ groups were greater. Compared with that of the LPS group, the OD value of the LPS + 10^− 8^ mol/L + E_2_ group was significantly increased (*P* < 0.001) (Fig. 2 - C).

#### The combined effects of P_4_ and LPS on the proliferation of mouse oviduct epithelial cells

Compared with those of the LPS group, the OD values of the P_4_ groups were greater. Compared with that of the LPS group, the OD value of the LPS + 10^− 7^ mol/L + P_4_ group was significantly increased (*P* < 0.001) (Fig. 2 - D).

### RT‒qPCR test results

**Fig. 3:**
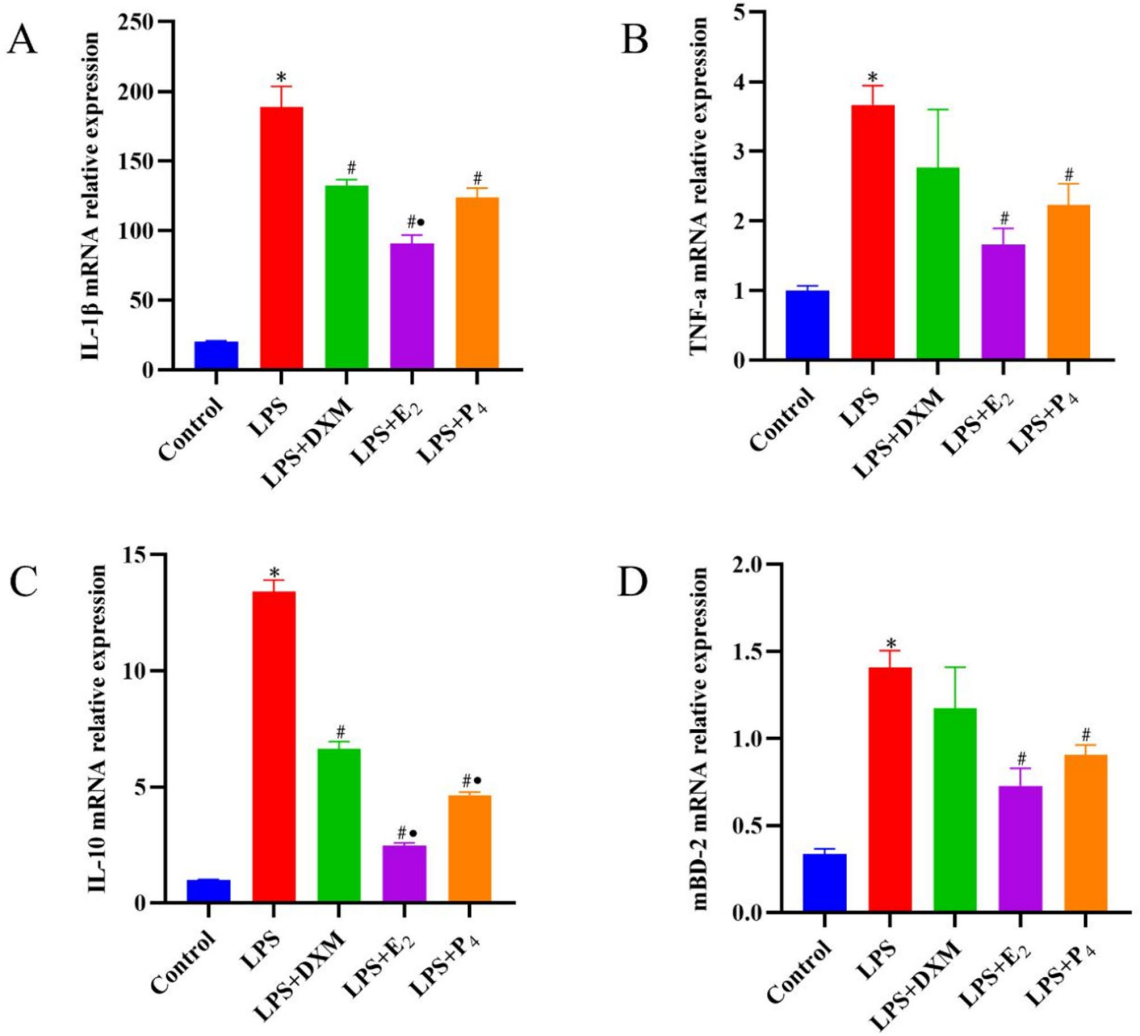
RT‒qPCR detection of the expression of IL-1β, TNF-α, IL-10, and mBD-2 in each group. **A**: RT‒qPCR detection of the expression of IL-1β in each group. **B**: RT‒qPCR detection of the expression of TNF-α in each group. **C**: RT‒qPCR detection of the expression of IL-10 in each group. **D**: RT‒qPCR detection of the expression of mBD-2 in each group. **Note**: *P < 0.05 compared with the control group; #*P* < 0.05 compared with the LPS group; •*P* < 0.05 compared with the DXM group

Compared with those in the control group, the mRNA expression levels of IL-1β, TNF-α, IL-10, and mBD-2 in the LPS group were significantly greater (*P* < 0.05). However, compared with the LPS group, the LPS + E_2_ and LPS + P_4_ groups presented significantly lower expression levels of IL-1β, TNF-α, IL-10, and mBD-2 (*P* < 0.05) (Fig. 3).

### ELISA test results

**Fig. 4:**
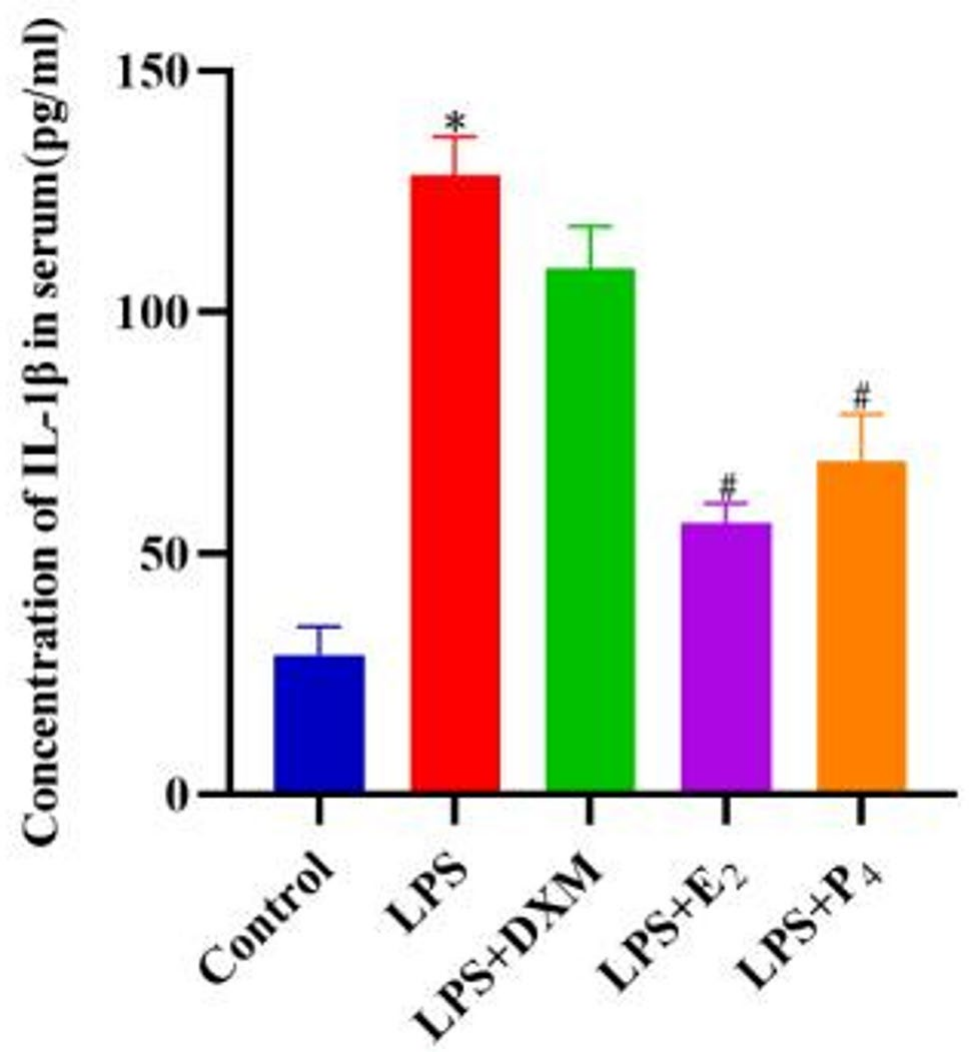
ELISA detection of IL-1β levels in each group. **Note**: *P < 0.05 compared with the control group; #*P* < 0.05 compared with the LPS group; •*P* < 0.05 compared with the DXM group

Compared with that in the control group, the level of the inflammatory factor IL-1β in the LPS group was significantly greater (*P* < 0.05). Compared with that in the LPS group, the level of IL-1β in the LPS + DXM group tended to decrease, but the difference was not statistically significant (*P* > 0.05). Compared with that in the LPS group, the level of IL-1β significantly decreased in the LPS + E_2_ and LPS + P_4_ groups (*P* < 0.05) (Fig. 4).

### Western blot results

#### NF-κB signaling pathway

**Fig. 5:**
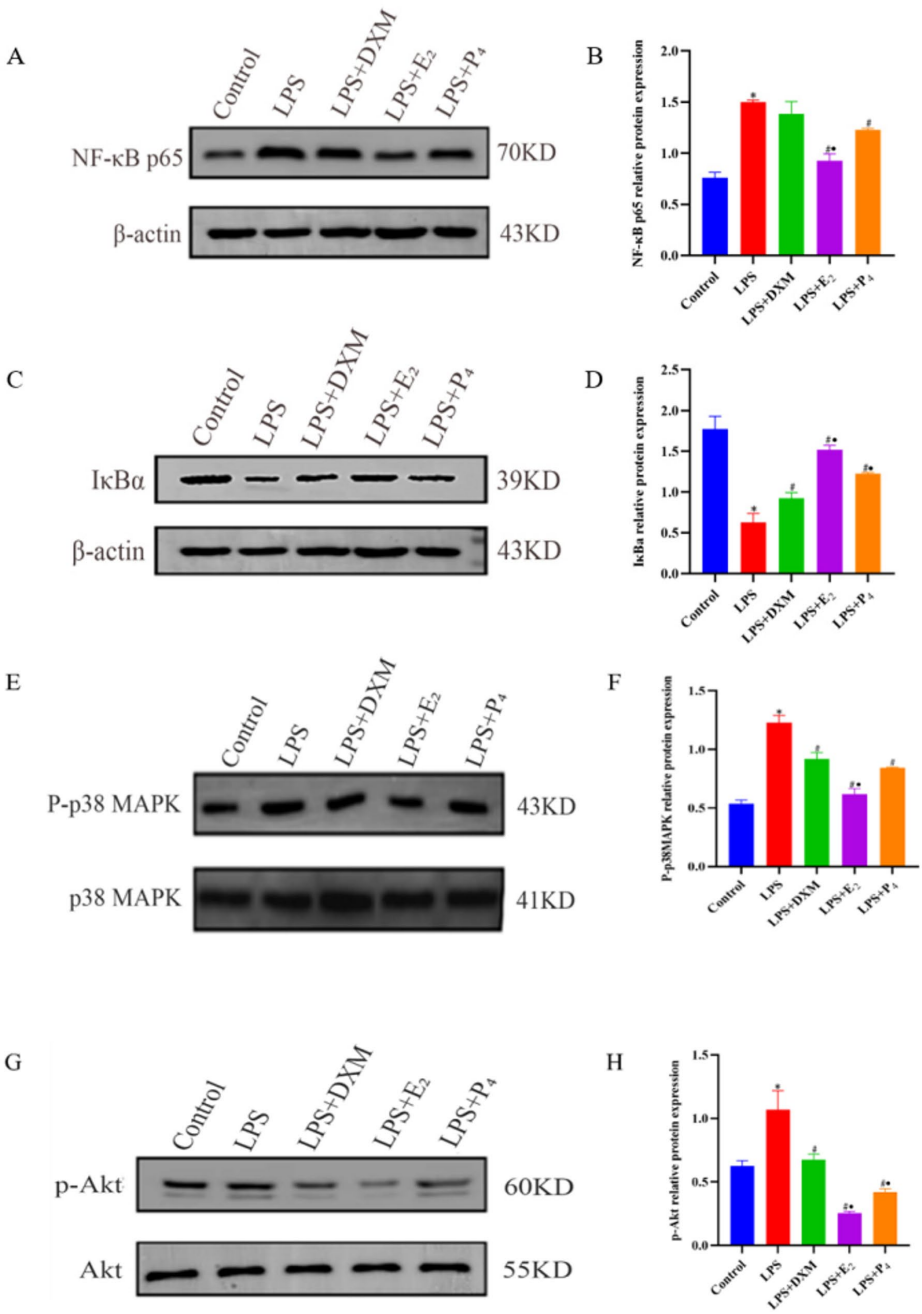
Western blot detection of protein in each group. **A** and **B**: Western blotting was used to detect the protein expression of mOEC NF-κB p65 in each group. **C** and **D**: Western blotting was used to detect the protein expression of mOEC IκBα in each group. **E** and **F**: Western blotting was used to detect the expression of the mOEC P-p38 MAPK protein in each group. **G** and **H**: Western blot detection of p-Akt protein expression in each group. ***Note***: *P < 0.05 compared with the control group; #*P* < 0.05 compared with the LPS group; •*P* < 0.05 compared with the DXM group

Compared with that in the control group, NF-κB p65 protein expression in the LPS group was significantly greater (*P* < 0.05). Compared with that in the LPS group, NF-κB p65 protein expression in the LPS + P_4_ and LPS + E_2_ groups was significantly lower (*P* < 0.05). Compared with that in the LPS group, NF-κB p65 protein expression in the DXM group tended to decrease but was not statistical significant (*P* > 0.05) (Fig. 5 - A and B).

Compared with that in the control group, the protein expression of IκBα in the LPS group was significantly lower (*P* < 0.05). Compared with that in the LPS group, the protein expression of IκBα in the LPS + DXM, LPS + E_2_, and LPS + P_4_ groups was significantly greater (*P* < 0.05). Compared with that in the LPS + DXM group, the protein expression of IκBα in the LPS + E_2_ group and the LPS + P_4_ group was significantly greater (*P* < 0.05). Compared with that in the LPS + E_2_ group, the protein expression of IκBα in the LPS + P_4_ group was significantly lower (*P* < 0.05) (Fig. 5 - C and D).

### MAPK test results

Compared with that in the control group, the P-p38 protein level in the LPS group was significantly greater (*P* < 0.05). Compared with that in the LPS group, the P-p38 protein level in the LPS + DXM, LPS + E_2_, and LPS + P_4_ groups was significantly lower (*P* < 0.05). Compared with that in the LPS + DXM group, the P-p38 protein level in the LPS + E_2_ group was significantly lower (*P* < 0.05). Compared with that in the LPS + DXM group, the protein expression of P-p38 in the LPS + P_4_ group was lower, but the difference was not statistically significant (*P* > 0.05) (Fig. 5 - E and F).

### PI3K/Akt test results

The protein expression of p-Akt in the LPS group was significantly greater than that in the control, LPS + DXM, LPS + E_2_, and LPS + P_4_ groups (*P* < 0.05). Compared to the LPS + DXM group, both the LPS + E_2_ and LPS + P_4_ groups presented a decreasing trend in p-Akt protein expression, with a significant decrease in the LPS + E_2_ group (*P* < 0.05). The decrease in p-Akt protein expression in the LPS + P_4_ group was not statistically significant (*P* > 0.05). Compared with the LPS + P_4_ group, the LPS + E_2_ group presented a decrease in p-Akt protein, but the difference was not statistically significant (*P* > 0.05) (Fig. 5 - G and H).

### HE staining results

**Fig. 6:**
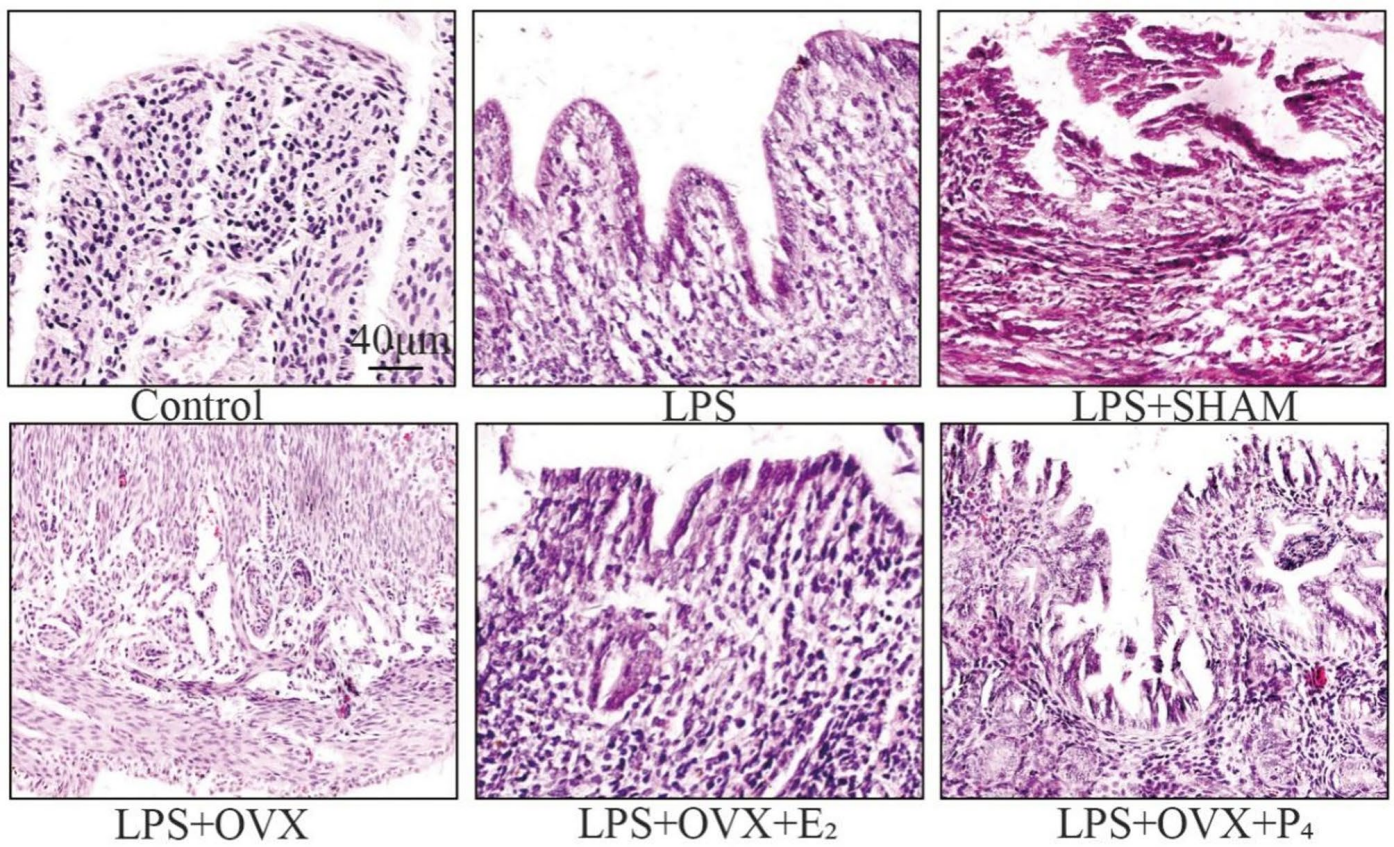
Histopathological changes in the fallopian tube tissues of mice in each group

As shown in Figs. 3, 4 and 5, in the control group, the fallopian tube wall mucosa was intact, with a clear structure, smooth lumen, and no accumulation of inflammatory cells. In the LPS group, there was mild inflammation in different areas of the fallopian tube, with slight disorganization and mild expansion of the epithelial and submucosal layers; in the LPS + SHAM group, mucosal shedding was observed. Moreover, in the LPS + OVX group, many neutrophils infiltrated the mucosa and muscle layers, causing flattening and a reduction in the number of mucosal folds accompanied by interstitial fibrosis. In the LPS + OVX + E_2_ group, the fallopian tube tissue structure in the mice was almost completely intact, with only slight fallopian tube mucosal lesions and a small amount of inflammatory cell infiltration. In the LPS + OVX + P_4_ group, the pathological changes were significantly ameliorated (Fig. 6).

### Immunohistochemistry results

#### Expression of IL-10

**Fig. 7:**
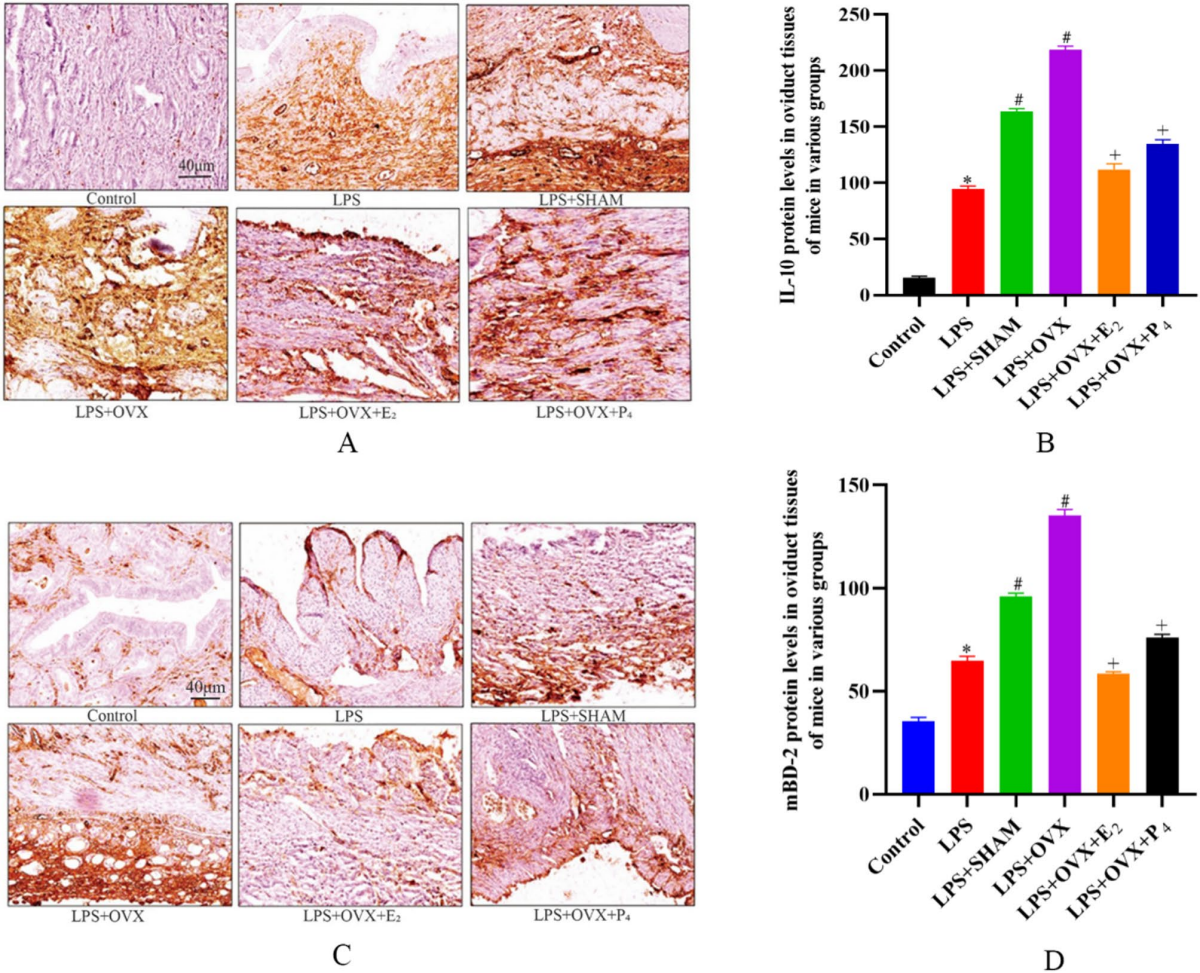
Effects of E_2_ and P_4_ on the expression of IL-10 and mBD-2 in the fallopian tube tissues of tubal inflammation model mice. **A** and **B**: Effects of E_2_ and P_4_ on the expression of IL-10 in the fallopian tube tissues of tubal inflammation model mice. **C** and **D**: Effects of E_2_ and P_4_ on the expression of mBD-2 in the fallopian tube tissues of salpingitis model mice. **Note**: Compared with the control group, *P < 0.05; compared with the LPS group, #*P* < 0.05; compared with the LPS + OVX group, +*P* < 0.05

The immunohistochemistry results revealed that the control group had essentially no staining, whereas the number of IL-10-positive cells and brown deposits in the fallopian tubes of the mice in the LPS, LPS + SHAM, and LPS + OVX groups were increased. In contrast, the numbers of IL-10-positive cells and brown deposits decreased in the LPS + OVX + E_2_ group and the LPS + OVX + P_4_ group (Fig. 7 - A).

The total score of IL-10 in the control group of mice was 0.75, that in the LPS group was 6.0, that in the LPS + SHAM group was 7.0, that in the LPS + OVX group was 8.25, that in the LPS + OVX + E_2_ treatment group was 3.0, and that in the LPS + OVX + P_4_ group was 6.25.

Compared with that in the control group, the expression of IL-10 in the fallopian tube tissues of the LPS, LPS + SHAM, and LPS + OVX groups was significantly greater (*P* < 0.05). Compared with that in the LPS + OVX group, the expression of IL-10 in the LPS + OVX + E_2_ and LPS + OVX + P_4_ groups was significantly lower (*P* < 0.05) but still significantly greater than that in the control group (*P* < 0.05) (Fig. 7 - B).

#### Expression of the mBD-2 protein

The immunohistochemistry results revealed that the control group had basically no staining, whereas the LPS, LPS + SHAM, and LPS + OVX groups of mice presented many mBD-2-positive cells and brown deposits in the fallopian tubes. The LPS + OVX + E_2_ group and the LPS + OVX + P_4_ group had a small number of mBD-2-positive cells and brown deposits (Fig. 7 - C).

The total score of mBD-2 in the control group was 0.35, that in the LPS group was 3.25, that in the LPS + SHAM group was 4.5, that in the LPS + OVX group was 8.0, that in the LPS + OVX + E_2_ group was 1.5, and that in the LPS + OVX + P_4_ group was 2.25.

Compared with that in the control group, the protein expression of mBD-2 in the fallopian tube tissue sections was significantly greater in the LPS, LPS + SHAM, and LPS + OVX groups (*P* < 0.05). Compared with that in the LPS + OVX group, the expression of mBD-2 was significantly lower in the LPS + OVX + E_2_ group and the LPS + OVX + P_4_ group after treatment (*P* < 0.05) but was still significantly greater than that in the control group (*P* < 0.05) (Fig. 7 - D).

## Discussion

Estrogen (E_2_) and progesterone (P_4_), key steroid hormones secreted by the ovaries, play indispensable roles in the body, finely regulating a wide range of physiological processes from the development of the reproductive system to maintaining internal homeostasis. Moreover, they exhibit profound effects and show extraordinary potential in preventing and treating various diseases [14]. Previous studies have shown that E_2_ and P_4_ are at high physiological concentrations have anti-inflammatory effects [15]. Studies have revealed that there are high concentrations of E_2_ and P_4_ in umbilical cord blood, which can effectively inhibit the production of cytokines by cord blood mononuclear cells (CBMCs) and newborn mononuclear cells, similar to the effects of hydrocortisone. The concentration of E_2_ in umbilical cord blood ranges from 2 µM to 150 µM, and the concentration of P_4_ in umbilical cord blood ranges from 1 µM to 5 µM [16]. Therefore, the carefully selected concentration ranges of E_2_ and P_4_ in this study were based on their inherent biological activity and therapeutic potential.

Under LPS stimulation, the secretion of proinflammatory cytokines increases sharply. Therefore, anti-inflammatory agents are commonly used as treatments [17]. In this study, the effects of E_2_ and P_4_ on the expression of IL-1β, TNF-α, IL-10, and mBD-2 in LPS-stimulated fallopian tube epithelial cells, as well as the potential mediation of postinjury salpingitis through the NF-κB, MAPK, and PI3K/Akt pathways, were investigated. LPS upregulated the expression of IL-1β, TNF-α, IL-10, and mBD-2 in fallopian tube epithelial cells, while E_2_ and P_4_ dose-dependently downregulated the expression of IL-1β, TNF-α, IL-10, and mBD-2. Furthermore, E_2_ and P_4_ inhibited LPS-induced NF-κB pathway activation by reducing IκBα phosphorylation and p65 nuclear translocation. E_2_ and P_4_ also decreased the phosphorylation of p38 MAPK in LPS-treated fallopian tube epithelial cells.

Akt is an important target signaling molecule in the downstream signaling pathway of PI3K [18]. The PI3K/Akt pathway has cell type specificity and depends on the stimulus applied [19]. Therefore, PI3K/Akt may exhibit proinflammatory or anti-inflammatory characteristics depending on the context. Research has shown that Akt can phosphorylate IκBα and activate the IKK complex, leading to the translocation of NF-κB from the cytoplasm to the nucleus, thereby regulating the synthesis and release of inflammatory factors [18]. Many studies have explored the therapeutic potential of targeting the NF-κB and MAPK pathways in tubal diseases [20–22]. The PI3K/Akt pathway is involved in multiple pathological conditions associated with epithelial cells, such as epithelial ovarian cancer, mammary epithelial cell inflammation, airway inflammation in asthma models, and airway hyperresponsiveness [23–25]. Moreover, the PI3K/Akt pathway has been shown to regulate the survival of various cells, including epithelial cells [26]. The pathway was also found to regulate epithelial-mesenchymal transition(EMT) [27]. Therefore, the PI3K/Akt signaling pathway may also be involved in the inflammatory response, fibrosis, and development of epithelial ovarian cancer in the fallopian tubes.

In addition, this study established a mouse model of oviduct inflammation. The study revealed that, after the mice in the LPS group were intraperitoneally injected with 50 µL of LPS (1 mg/mL, dissolved in sterile PBS) for 24 h, HE-stained tissue sections from each group were observed to confirm the successful establishment of oviduct inflammation in the mice. Previous studies have reported that E_2_ can effectively inhibit colon epithelium inflammation and inflammation-related colon tumor formation in mice [28–30].Additionally, P_4_ can effectively inhibit endometritis in cows and brain inflammation in model mice [31–33]. However, no studies have reported the effects of E_2_ and P_4_ in a mouse model of oviduct inflammation.

Interestingly, in this study, E2 and P4 not only reduced the expression of inflammatory factors but also decreased the expression of the anti-inflammatory factor IL-10. In previous studies exploring anti-inflammatory mechanisms, both increases [34–36] and decreases [37] in the levels of anti-inflammatory factors relative to reductions in the levels of inflammatory factors have been reported. On the basis of the results of this study and comparisons with those of previous studies, the significant inhibition of IL-10 by E2 and P4 is likely related to signaling pathways such as the NF-κB pathway and is also associated with feedback stimulation from inflammatory factors and a reduced demand for anti-inflammatory responses due to a weakened inflammatory environment. However, further experiments are needed to confirm these results.

In this study, we comprehensively evaluated the anti-inflammatory effects of E_2_ and P_4_ on LPS-induced mouse oviduct inflammation through in vivo and in vitro experiments. In the in vitro experiments, RT‒qPCR analysis revealed that the mRNA expression of IL-1β, TNF-α, IL-10, and mBD-2 was significantly increased in the LPS group, whereas the expression of these inflammatory factors was significantly inhibited in the LPS + E2 and LPS + P4 groups, suggesting that E_2_ and P_4_ can significantly suppress the expression of inflammatory factors induced by LPS. The ELISA results revealed that IL-1β levels were significantly elevated in the LPS group but were significantly inhibited in the LPS + E_2_ and LPS + P_4_ groups, indicating that E_2_ and P_4_ can significantly suppress the release of IL-1β induced by LPS. Western blot analysis revealed that the expression of NF-κB, MAPK, and PI3K/Akt pathway members was upregulated in the LPS group but significantly downregulated in the LPS + E_2_ and LPS + P_4_ groups compared with the LPS group, suggesting that E_2_ and P_4_ can downregulate inflammatory mediator expression in mouse oviduct epithelial cells induced by LPS by inhibiting the NF-κB, MAPK, and PI3K/Akt pathways. In the in vivo experiments, the HE staining results demonstrated that both E_2_ and P_4_ effectively alleviated the pathological damage caused by LPS. Furthermore, immunohistochemical analysis revealed that E_2_ and P_4_ significantly downregulated the expression levels of the inflammatory markers IL-10 and mBD-2 during inflammation.

Clinically, menopausal women are more prone to various types of reproductive system inflammation due to a decrease in estrogen levels. Estradiol and progesterone are the two most important hormones among female estrogens and have multiple biological functions. In this study, inflammation in mouse fallopian tube epithelial cells was induced via the use of LPS to simulate the inflammatory response in women’s fallopian tubes. In vivo and in vitro experiments demonstrated that estradiol and progesterone can effectively inhibit LPS-induced inflammatory responses, providing new ideas and methods for clinical treatment and subsequent related experiments.

## Conclusion

The results of this study indicate that estrogen and progesterone have a protective effect on LPS-induced mouse salpingitis, which may be attributed to the inhibition of inflammatory responses. The mechanism may be related to the inhibition of the NF-κB, MAPK, and PI3K/Akt signaling pathways and the regulation of the levels of the inflammatory factors IL-1β, TNF-α, IL-10, and mBD-2. On the basis of these experimental results, it is speculated that estrogen and progesterone have certain alleviating effects on inflammation.

## Electronic supplementary material

Below is the link to the electronic supplementary material.

Supplementary Material 1

Supplementary Material 2

## Data Availability

No datasets were generated or analysed during the current study.
