# Supplementary material for: Inhibition of LPS-induced inflammation and signaling pathways in fallopian tube epithelial cells by estrogen and progesterone
Source: BMC Vet Res. 2025 Jul 2;21:421. doi: 10.1186/s12917-025-04874-x (PMC12219987; doi:10.1186/s12917-025-04874-x)
Supplement: Supplementary file 1 — Supplementary Material 1 [file 12917_2025_4874_MOESM1_ESM.pdf]

The gels images of WB

NF-κB P65

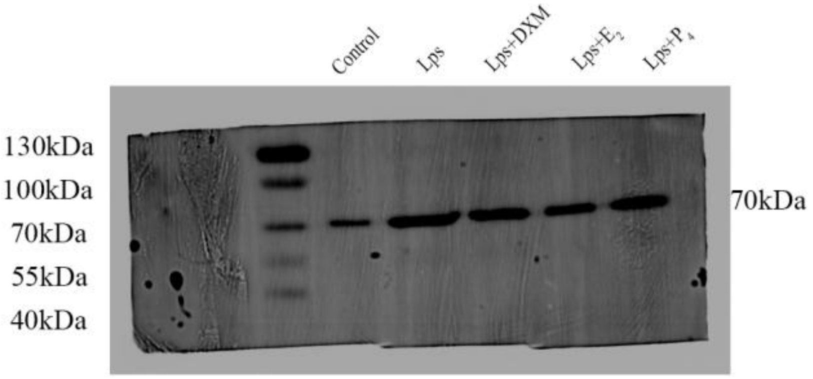

β-actin

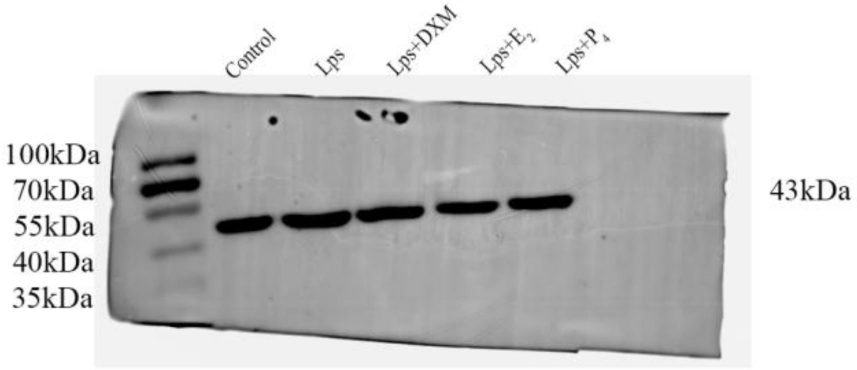

IκBα

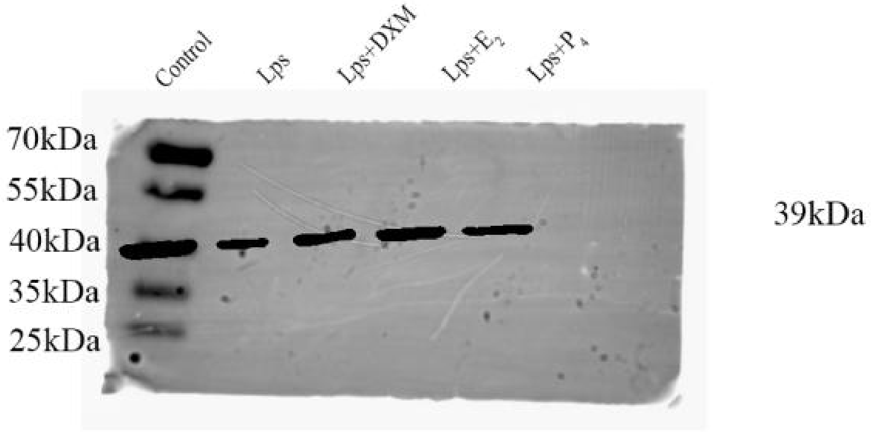

β-actin

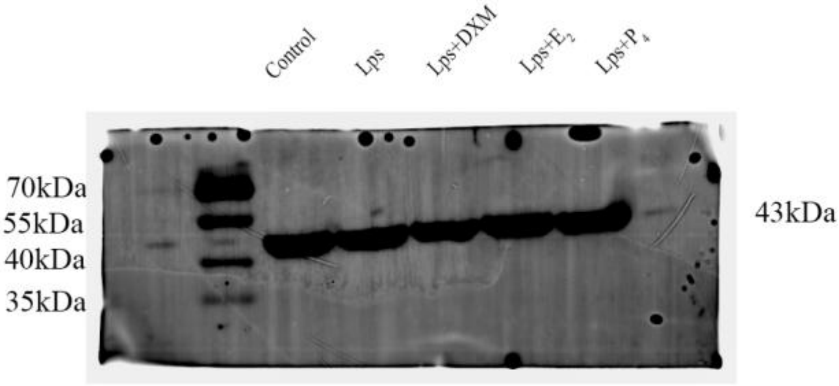

P-P38 MAPK

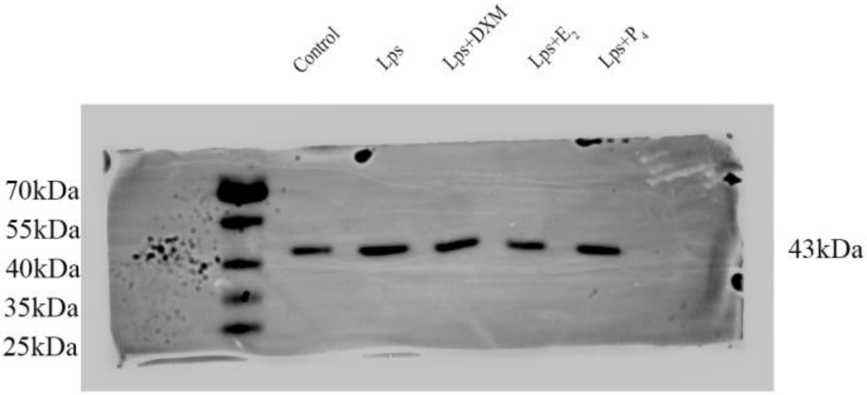

P38 MAPK

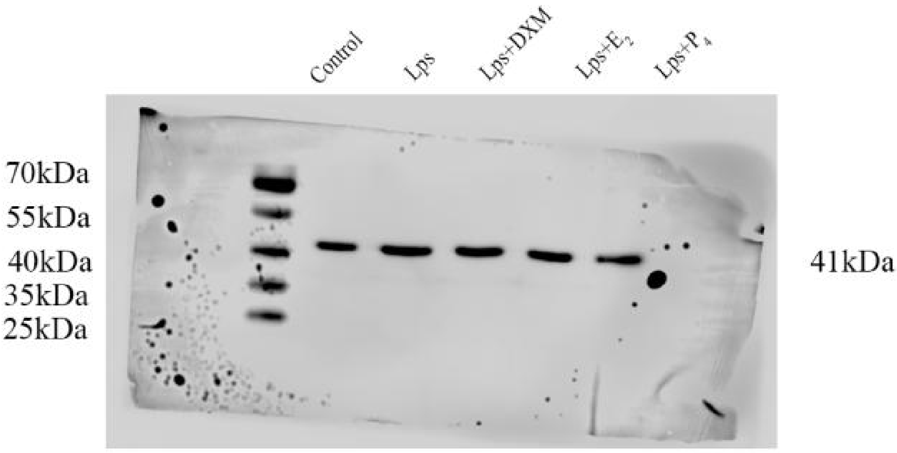

P-Akt

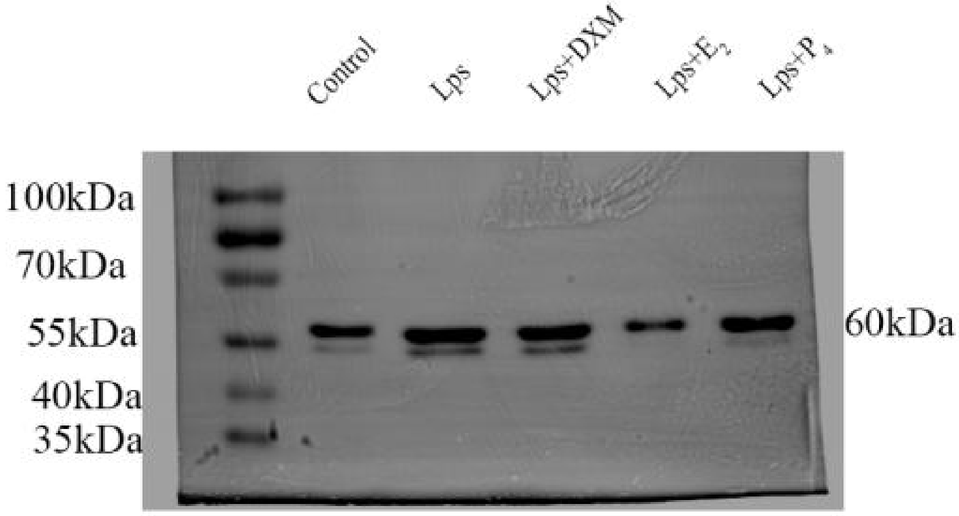
Control
Lps
Lps+DXM
Lps+E_2
Lps+P_4
100kDa
70kDa
55kDa
40kDa
35kDa
60kDa

Akt

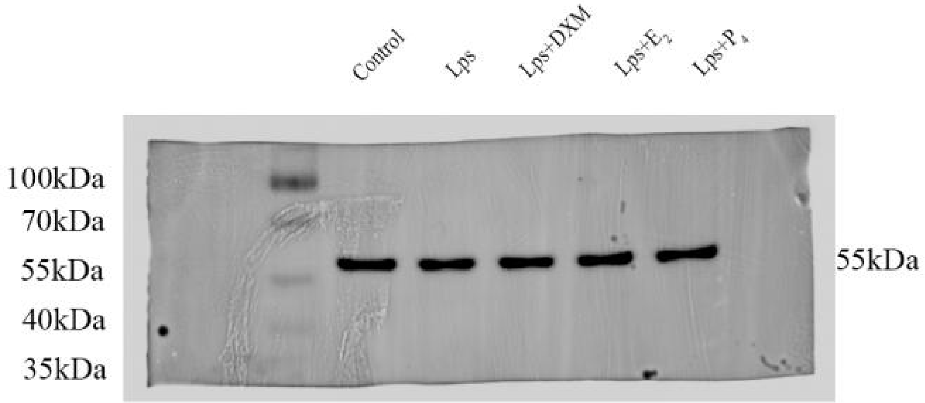
Control
Lps
Lps+DXM
Lps+E_2
Lps+P_4
100kDa
70kDa
55kDa
40kDa
35kDa
55kDa
